# Social Responsiveness as a Mediator in Adapted Cognitive Behavioral Therapy for Autistic Youth with Maladaptive and Interfering Anxiety

**DOI:** 10.1007/s10803-025-06866-0

**Published:** 2025-05-07

**Authors:** Jeffrey J. Wood, Eric A. Storch, Katherine Sung, Karen S. Wood, Philip C. Kendall

**Affiliations:** 1 Department of Education, University of California, Los Angeles, CA USA; 2 Department of Psychiatry, University of California, Los Angeles, CA USA; 3 Department of Psychiatry and Behavioral Sciences, Baylor College of Medicine, Houston, TX USA; 4 Department of Psychology and Neuroscience, Temple University, Philadelphia, PA USA; 5 UCLA-Moore Hall, 405 Hilgard Ave, Los Angeles, CA 90095 USA

**Keywords:** Autism, Cognitive-behavioral therapy, Anxiety, Social responsiveness

## Abstract

Numerous adaptations to interventions have been included in cognitive behavioral therapy (CBT) for autistic youth. This study examines the degree to which CBT adapted to the social needs of autistic youth confers significant benefit by promoting social responsiveness. A secondary analysis was conducted on a multisite randomized clinical trial (Wood et al. in JAMA Psychiatry 77:474–483, 2020) comparing adapted CBT with standard-of-practice CBT and treatment-as-usual (TA). Autistic youth (*N* = 167; aged 7–13) with maladaptive and interfering anxiety participated. The adapted CBT (BIACA) uses a modular format, with an emphasis on supplementing common CBT practice elements (reframing and graded exposure) with social skill supports. The primary outcome measure was the Pediatric Anxiety Rating Scale. Social responsiveness was assessed with the Social Responsiveness Scale, Second Edition. Participants’ general mental health was assessed as a secondary outcome using the Brief Problem Checklist. Mediation was tested using the SPSS PROCESS macro. Analyses suggested that the effect of adapted CBT on anxiety was mediated by its effects on social responsiveness, with a statistically significant indirect effect. Youth randomly assigned to adapted CBT exhibited better overall mental health at posttreatment compared to those randomized to the other conditions, and this effect was also mediated by improved social responsiveness. CBT adapted to address some of the social needs of autistic youth may enhance mental health outcomes by supporting social responsiveness, perhaps increasing the ease and effectiveness with which some youth can navigate potentially stressful situations such as entering and participating in group activities.

## Introduction

Several adapted cognitive behavioral therapy (CBT) programs have been developed for the mental health and autism-related needs of individuals on the autism spectrum over the past 20 years and have included variations in session format (e.g., emphasis on visual learning, caregiver involvement), content (e.g., emphasis on social skills), or both (see Walters et al., 2016). In a large, multisite trial comparing adapted CBT to standard-of-practice CBT and treatment as usual (TAU) for autistic youth with maladaptive and interfering anxiety (*N* = 167), both CBT conditions were associated with positive treatment response for most participants, with adapted CBT exhibiting additional positive impact for the primary outcome measure, independent evaluator ratings of anxiety (Wood et al., 2020). Which adaptations confer the clinical benefit in adapted CBT, however, remain unknown (e.g., Weston et al., 2016).

Challenges with (and different patterns of) social responsiveness are defining characteristics of autism. Social responsiveness has been defined as reciprocal social behavior, such as responding to others and playing cooperatively (e.g., Constantino et al., 2000). Less social responsiveness has been found to be associated with higher anxiety and other mental health needs in numerous quantitative (e.g., Ratcliffe et al., 2015) and qualitative studies (e.g., Cresswell et al., 2019; Milner et al., 2019; Tierney et al., 2016) in samples of autistic youth and adults. Using social communication skills that engender positive responses from others might, over time, facilitate an individual’s expectations of their likelihood of goal attainment in socially embedded contexts (e.g., school, sports, group play) that correspondingly reduce anxiety about rejection and failure as well as negative self- and other-appraisals in these contexts. Notably, some behavioral interventions that have focused primarily on social skills have had secondary positive impacts on social anxiety (cf. Factor et al., 2022).

The present study employed the experimental therapeutics framework (Insel, 2015) to discern whether a CBT program that was adapted to the social needs of autistic youth confers clinical benefit by promoting social responsiveness. The experimental therapeutics framework highlights the need to establish mediators of an intervention so that future refinements of the intervention (and future novel interventions) may focus on enhancing proximal treatment effects that mediate desired outcomes.

A common emphasis within CBT protocols adapted for autistic youth has been support for social skills and friendship (e.g., White et al., 2013). Examples of such adaptations use CBT to address perspective taking, joining group activities, starting and maintaining conversations, and hosting one-on-one get-togethers. In research on adapted CBT protocols that emphasize social skill supports, several efficacy studies with a variety of comparison groups have found a positive impact of adapted CBT on various measures of social responsiveness (i.e., caregiver ratings of social responsiveness, school observations of appropriate peer engagement by independent evaluators, and observations of appropriate social engagement in the home setting by independent evaluators; Storch et al., 2013, 2015; White et al., 2013; Wood et al., 2009, 2015, 2020, 2022). Within the experimental therapeutics framework, this set of findings has established a probable impact of adapted CBT on a target mechanism (i.e., social responsiveness). However, whether this target serves as a mediator of treatment outcome (e.g., anxiety) has not been tested.

A common understanding of the effects of standard-of-practice CBT on maladaptive and interfering anxiety is that it operates through either (a) extinction or new learning (i.e., exposure), (b) change in coping efficacy (e.g., Kendall et al., 2016) or (c) facilitating the activation of benign schemata instead of irrational and catastrophic schemata in feared situations (e.g., Brewin, 2006, 2015). This understanding assumes that feared situations causing interfering anxiety are essentially benign in character (e.g., would not result in feared outcomes if the individual would approach and engage). However, entering a challenging social situation (e.g., joining a game with rules without understanding how to play or cooperate with teammates) without requisite skills can result in failure, with the potential for social reprobation (e.g., criticism or exclusion) and increased anxiety. Because autism is associated with social challenges, the skills needed to succeed in feared or avoided social situations cannot be assumed. Hence, a CBT intervention model that addresses social skills as a means of altering objectively stressful social circumstances that have triggered anxiety might enhance treatment outcomes in comparison with standard-of-practice CBT. Additionally, for autistic individuals, developing relevant social skills could hypothetically serve as a conduit for additional gains in broader aspects of mental health; for example, greater social responsiveness could facilitate cooperation with others (as opposed to defiance and conflict) and feelings of inclusion and acceptance (as opposed to loneliness and negative self-appraisal).

The present study examined the hypothesis that increased social responsiveness serves as a mediator of change in the effects of adapted CBT on maladaptive and interfering anxiety. In testing this hypothesis, standard-of-practice CBT and TAU, two interventions that did not significantly impact social responsiveness in the clinical trial referenced above (Wood et al., 2020), served as the reference condition. A secondary study aim was to examine the effect of adapted CBT on an index of children’s general mental health via increased social responsiveness.

## Method

This study is a secondary analysis of data from a clinical trial (Wood et al., 2020) conducted at three research centers in the U.S. (*n*s = 64, 61, and 42). The sample consisted of 167 autistic youth (ages 7–13 years) with maladaptive and interfering anxiety (with Pediatric Anxiety Rating Scale [PARS; Research Units on Pediatric Psychopharmacology Anxiety Study Group, 2002] total summed scores greater than or equal to 17). IRBs at each site approved this study. Table 1 presents summary information on the participants.

**Table 1:** Demographic and clinical characteristics for the three conditions

Characteristics	Participants, no./Total no. (%)		
	Adapted CBT	Standard CBT	TAU
Child’s gender (female)	21/68 (31)	10/59 (17)	0/18 (0)
Child’s ethnic^a^ background			
Latino/a	9/68 (13)	10/58 (27)	3/18 (17)
Child’s racial^a^ background			
African American/African	6/68 (9)	1/58 (2)	3/18 (17)
Asian/Pacific Islander	4/68 (6)	3/58 (5)	1/18 (6)
Caucasian	52/68 (77)	48/58 (83)	12/18 (67)
Native American or Alaskan	2/68 (3)	1/58 (2)	0/18 (0)
Multiracial	4/68 (6)	5/58 (9)	2/18 (11)
Total household income < $40,000	14/68 (21)	11/58 (19)	5/18 (28)
Parents currently married	53/68 (78)	43/58 (74)	11/18 (61)

The sample sizes vary within group because some demographic data was not provided by some families

*TAU* Treatment as Usual

^a^ Race and ethnicity were queried in the same section of the survey, leading many families to report on race *or* ethnicity, but not both, and in limited detail

This study recruited autistic children and youth from local clinics and referral networks at each site. Potentially eligible children were phone-screened with their caregivers, followed by in-person diagnostic evaluations (conducted by reliable independent evaluators; IEs) and research assessments prior to randomization. Eligibility criteria included receiving scores above the clinical cutoff for the autism spectrum on the Autism Diagnostic Observation Schedule-2 (Lord et al., 2012) and the Childhood Autism Rating Scale Second Edition (Schopler et al., 2010) as well as a clinical diagnosis of autism; estimated full-scale IQ scores from an abbreviated Wechsler Intelligence Scale for Children-IV (Wechsler, 2003) assessment above 68 (i.e., 70, plus or minus the SEM); and PARS scores as noted above. Participants were excluded if they were receiving concurrent therapy, social skills training with homework, or behavioral interventions (e.g., applied behavior analysis). Families had the option of discontinuing such services to enroll. However, participants were allowed to continue with academic tutoring, occupational therapy, speech therapy, or school counseling (no more than 60 min per week), school aides, and social skills training groups that did not include homework. Concurrent psychiatric medications were allowed as long as children were on a stable dose at the time of baseline assessment and did not plan to change dose during the course of the trial.

Assessments and CBT interventions took place in the university settings associated with the three research sites. TAU was delivered in the community-based settings selected by each family. Nineteen graduate students and postdoctoral fellows served as therapists and received eight hours of training in the treatment protocols. They also reviewed the treatment manuals and participated in weekly supervision meetings led by a licensed psychologist. Children were matched with therapists based on therapist availability. All therapists were trained to deliver both CBT interventions, and provided the CBT condition that each family was randomized to.

### Measures

The primary outcome measure was the PARS, an IE-administered scale assessing anxiety symptoms and associated impairment over the past week. The seven PARS Severity Scale item scores range from 0 to 5, with higher scores reflecting more maladaptive and interfering anxiety. High inter-rater reliability (*r* > 0.80) for PARS ratings in this sample is reported in Wood et al. (2020). To maintain consistency with conventions established in Wood et al. (2020), hypotheses related to the PARS are tested using mean item ratings on the PARS Severity Scale items (mathematically equivalent to summed scores for the statistical tests employed in this study). PARS scores from baseline and acute post-treatment were used for this study.

The Social Responsiveness Scale-2 (SRS; Constantino & Gruber, 2012) was also administered at baseline and acute post-treatment. The SRS is a parent-report measure of autism-related behaviors, with 65 items rated on a 0–3 Likert-type scale; the factor-analytically derived Social Communication/Interaction (SCI) subscale was employed for this study (following Wood et al., 2020). High internal consistency (alpha > 0.80) for this subscale in this sample at both timepoints is reported in Wood et al. (2020). Higher scores reflect more frequent manifestations of social challenges and less responsiveness; scores are the average item value of the SRS-SCI subscale.

The Brief Problem Checklist (BPC) is an item-response theory-derived measure of children’s mental health, broadly construed, based on the Child Behavior Checklist (CBCL; Achenbach & Rescorla, 2011) item set and derived to maximize sensitivity to treatment effects (Chorpita et al., 2010). The original BPC item set is comprised of 12 items from the CBCL representing traits across two broad domains of children’s mental health—internalizing and externalizing (e.g., worries, self-conscious, tantrums, argues, stubborn, feels worthless). Items are rated on a 3-point Likert scale from 0 to 2, with higher scores indicating more problems. The original BPC collapsed two CBCL items (disobedience at home, disobedience at school) into one item; in the present study, this item was disaggregated back into the two separate items as used in the CBCL, for a total of 13 items. The BPC has excellent psychometric properties for the assessment of clinical change during intervention, including evidence of validity and reliability in autistic youth in previous samples as well as in the present sample (see Online Resource 1 for details). Scores are the sum of the 13 BPC items.

### Interventions

Participants were randomized using a computer-generated algorithm in a parallel study design with a 4.5:4.5:1 ratio to (a) standard-of-practice CBT (*Coping Cat*), (b) adapted CBT (Behavioral Interventions for Anxiety in Children with Autism; BIACA [Wood et al., 2020]), or, (c) TAU.

#### Standard-of-Practice CBT (Coping Cat)

Participants received 16 weekly 60-min sessions of the *Coping Cat* program delivered in a one-on-one format. *Coping Cat* has been found to be efficacious in trials of typically developing children and youth aged 7–13 years (e.g., Kendall et al., 2008). The main features of *Coping Cat* are: (a) recognizing anxious feelings and somatic reactions to anxiety, (b) identifying cognition in anxiety-provoking situations (i.e., expectations of threat), (c) developing a plan to cope (i.e., reappraisal), (d) imaginal and in vivo exposure tasks, and (e) reinforcement for effort. The treatment uses modeling, role-play, and contingent reinforcement. Specific homework tasks are assigned. Parent involvement in the treatment includes a regular check-in at the start of each session and two meetings with the therapist.

#### Adapted CBT (BIACA)

In BIACA, children received 16 weekly 90-min sessions delivered in a one-on-one format, with sessions split evenly between children and parents. BIACA employs a modular format guided by an algorithm to personalize treatment given the multifaceted clinical presentations in autistic youth. Modules include CBT strategies such as reappraisal and exposure (similar to Coping Cat), as well as social coaching on joining, initiating, and maintaining social interactions, play date hosting, self-presentation (including awareness of social expectations in specific situations), regulation of disruptive behavior, and self-care skills. Children’s interests (restricted or otherwise) are treated as an asset and incorporated into treatment to promote engagement. Motivational strategies pertaining to the acquisition and mastery of social skills are incorporated into relevant sessions, including discussing the child’s goals around friendship (who would they like to make closer friends with?) and using perspective taking exercises to align the child’s motivation to get along with others with how specific social responses (e.g., smiling, indifference) may be perceived. Further clinical description of BIACA and its treatment algorithm is provided in Wood et al. (2011). A training and clinician guidance app incorporating the algorithm has been developed for BIACA and related practices, available free of charge at meya.ucla.edu.

#### Treatment-as-Usual (TAU)

TAU participants continued in their usual services and were provided with referrals. No specific treatment recommendations were given. Families were permitted to choose or maintain any treatment approach for 4 months. The majority of families (71%) received psychosocial or psychiatric services for their autistic child during TAU (Wood et al., 2020), and nearly the same proportion of participants (65%) received psychotherapy or counseling specifically.

Evidence of high levels of treatment fidelity in the two CBT conditions is reported in Wood et al. (2020). The majority of children completed the intervention and returned for a posttreatment assessment in each condition (87% overall). The rates of study discontinuation between the three treatment conditions did not differ significantly.

### Data Analysis

The experimental therapeutics framework (Insel, 2015) was applied in the design of the analytic plan. In the primary report of the outcomes, adapted CBT outperformed standard-of-practice CBT and TAU on ratings of anxiety severity (PARS) and social responsiveness (SRS) at posttreatment (Wood et al., 2020). These findings establish the effect of an intervention (adapted CBT) on both a target (social responsiveness) and outcome (anxiety). However, the linkage between change in the target and change in the outcome (while controlling for condition) has not yet been examined; this linkage serves as a prerequisite for establishing a target variable (mediator) as a possible mechanism of action in the intervention’s effect on the outcome. The indirect effect of adapted CBT on maladaptive and interfering anxiety via social responsiveness was tested using the SPSS macro PROCESS (Hayes, 2022; model 4; seed = 30,802,022), which uses bootstrapping (5000 bootstrap samples) to estimate mediation effects. In this analysis, pre-post change scores were computed for SRS scores and PARS scores at the person level. Indicator variable coding was used in which adapted CBT (coded 0.5) was compared to standard-of-practice CBT and TAU (coded −0.5). PARS change scores served as the DV, the adapted CBT indicator variable was the IV, and SRS change scores served as the mediator. The indirect effect and a path model depicting the estimated effects are reported.

In secondary analyses, the effect of adapted CBT on broader mental health outcomes (BPC-Total scores) via social responsiveness (SRS) was also tested. First, a general linear mixed model (GLMM) with site as a random effect was estimated examining the fixed effect of adapted CBT on post-treatment BPC-Total scores (controlling for pre-treatment BPC-Total scores; cf. Wood et al., 2020). As with the primary outcome analyses (Wood et al., 2020), this model was estimated using HLM statistical software (Scientific Software International Inc.). Second, the SPSS PROCESS macro was used to test the indirect effect of adapted CBT on pre-post changes in BPC-Total scores via pre-post changes in SRS scores, parallel to the primary analysis for PARS outcome detailed above. All analyses were by the original assigned groups.

For all analyses, change scores are computed as posttreatment scores minus pretreatment scores. Thus, change scores with negative values indicate improvement on the PARS, SRS, and BPC.

## Results

Of the 167 children randomized in the primary clinical trial, 145 had sufficient data to be included in the present analyses; 68 randomized to adapted CBT, 59 randomized to standard-of-practice CBT, and 18 randomized to TAU. Table 1 presents demographic information for families included in this analysis. All dependent variables had skewness and kurtosis coefficients within normal limits (coefficients divided by their SEs were below 1.96).

### Primary Analysis

**Fig. 1:**
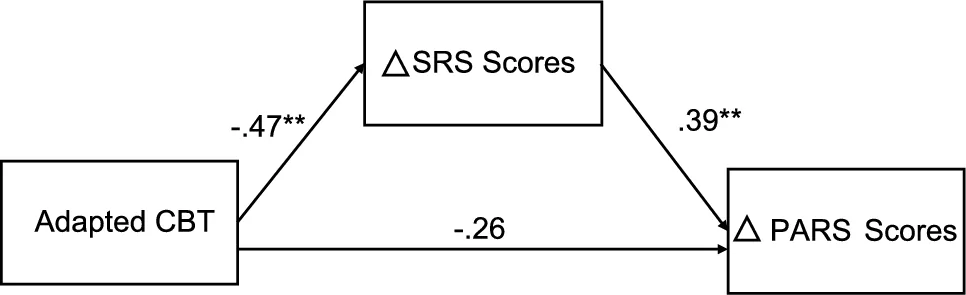
Regression-based path model of change in social responsiveness as a mediator of the linkage between adapted CBT and change in maladaptive and interfering anxiety. This figure provides standardized coefficients of a path analysis examining the effect of Adapted CBT (vs. standard-of-practice CBT and TAU) on Pediatric Anxiety Ratings Scale (PARS) change scores via its effect on Social Responsiveness Scale-2 (SRS) change scores. Negative change scores reflect clinical improvement on the SRS and the PARS. The indirect effect of Adapted CBT on change in PARS scores was −0.18 (*p* < 0.05)

Descriptive information on the outcome variables is provided in Table 2. Figure 1 presents standardized path coefficients for the mediation analysis (using the PROCESS macro) described above. The model supports a statistically significant mediated effect of adapted CBT on change in PARS scores via change in SRS scores. Adapted CBT was linked with greater improvement in SRS change scores, which, in turn, was linked with greater reductions in PARS change scores (while controlling for condition). The partially standardized indirect effect of adapted CBT on change in PARS scores was estimated to be −0.18 (SE = 0.08, 95% CI: −0.36, −0.05). This model supports the conclusion that some of the anxiety reduction associated with adapted CBT is partially mediated by increases in social responsiveness.

**Table 2:** Parameters for primary and secondary outcome measures for the three conditions at pre- and post-treatment

Scale	Adapted CBT		Standard CBT		TAU	
	Pre	Post	Pre	Post	Pre	Post
PARS Severity M	3.41	2.14	3.42	2.43	3.28	2.93
SD	0.49	0.91	0.45	0.70	.40	0.59
n	66	66	58	58	18	18
SRS-SCI M	1.54	1.31	1.58	1.47	1.50	1.55
SD	0.32	0.37	0.36	0.40	0.27	0.34
n	60	60	52	52	14	14
BPC-Total						
M	10.36	7.60	11.72	9.36	9.20	8.87
SD	4.98	4.70	4.66	4.62	3.99	3.94
n	64	64	54	54	15	15

*TAU* Treatment as Usual, *PARS* Pediatric Anxiety Rating Scale, *SRS SCI* SRS Social Communication/Interaction scale, *BPC* Brief Problem Checklist Total scale

### Secondary Analyses

Parameters from the GLMM used to test the impact of adapted CBT on BPC-Total scores are presented in Table 3. Children randomized to adapted CBT had statistically significantly lower post-treatment BPC-Total scores in comparison with children randomized to the other two conditions. Hence, caregiver ratings of a broad spectrum of mental health needs in autistic children exhibited greater reductions at post-treatment in the adapted CBT condition.

**Table 3:** Coefficients for GLMM model of the effect of adapted CBT (vs. standard CBT and TAU) on post-treatment BPC Total scores

Fixed effects	Estimate	*SE*	*p*
Intercept	1.08	0.68	0.254
Pre-treatment BPC total score	0.68	0.06	<.001
Adapted CBT	−1.10	0.55	0.049

Random effects	SD	Variance component	*p*
Level-1 residual	3.17	10.09	–
Intercept (site)	0.03	0.001	> 0.50

The effect of Adapted CBT (coded 0.5) was tested against the reference group (Standard-of-Practice CBT and TAU, coded −0.5)

**Fig. 2:**
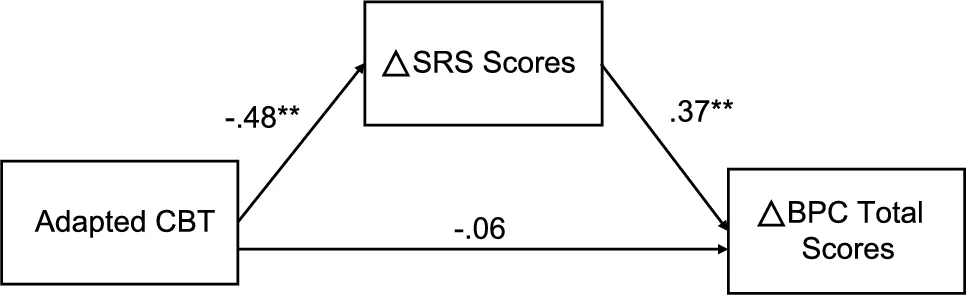
Regression-based path model of change in social responsiveness as a mediator of the linkage between adapted CBT and change in general mental health. This figure provides standardized coefficients of a path analysis examining the effect of Adapted CBT (vs. standard-of-practice CBT and TAU) on Brief Problem Checklist (BPC) Total change scores via its effect on Social Responsiveness Scale-2 (SRS) change scores. Negative change scores reflect clinical improvement on the SRS and the BPC. The indirect effect of Adapted CBT on change in BPC Total scores was −0.18 (*p* < 0.05)

In Fig. 2, standardized path coefficients are provided from a mediation analysis using the PROCESS macro in which the adapted CBT indicator variable served as the IV, SRS change scores served as the mediator, and BPC-Total change scores served as the DV. As with the PARS model, above, results indicate that adapted CBT was associated with greater pre- to post-treatment improvements in SRS scores, which in turn was linked with greater reductions in BPC-Total scores. The partially standardized indirect effect of adapted CBT on BPC-Total change scores was −0.18 (SE = 0.08; 95% CI: −0.35, −0.04). This model supports a statistically significant positive effect of adapted CBT on a broad measure of children’s mental health needs partially mediated by improvements in social responsiveness.

## Discussion

This study adopted the experimental therapeutics framework (Insel, 2015) to examine social responsiveness as a possible mediator of the effects of adapted CBT for autistic children and youth on intervention outcomes. Results supported the hypothesis that the greater reductions in maladaptive and interfering anxiety associated with adapted CBT (i.e., Wood et al., 2020) were partly mediated by greater improvements in social responsiveness over the course of the intervention. Furthermore, adapted CBT outperformed comparators on general mental health outcomes (beyond anxiety), with these effects also mediated by improvements in social responsiveness. Comparatively, change in anxiety for non-autistic youth was mediated by change in coping efficacy and change in self-talk in a large randomized controlled trial of CBT and medication (Kendall et al., 2016). CBT also positively impacts coping efficacy in autistic youth (Norris et al., 2023), and has recently been found to serve as a possible mediator (Wood et al., in press) for the youth on the autism spectrum in the present trial.

The experimental therapeutics framework (Insel, 2015) emphasizes the mechanisms underlying successful mental health treatments. Social responsiveness needs are an essentially universal feature of the autism spectrum, which was assessed in this study as a potential mechanism of change for a mental health intervention adapted for autistic children and youth. The linkage between social skills and mental health needs are well documented in both observational studies (e.g., Ratcliffe et al., 2015) as well as qualitative accounts of autistic individuals and stakeholders who have described the role of social challenges in emotional reactions ranging from anxiety and self-consciousness to low self-worth, anger, and despair (Cresswell et al., 2019; Milner et al., 2019; Tierney et al., 2016). It is perhaps not surprising that a notable, positive intervention impact on a target such as social skills would have the capacity to mediate additional benefit in mental health outcomes in a population experiencing social challenges. When children have repeatedly attempted to engage socially and have experienced failure, further efforts to join in the peer group (i.e., exposure therapy) without compensatory social skills might only further compound feelings of exclusion and dysphoria. Instead, social skill supports to facilitate positive peer engagement might facilitate the success of approach-oriented behavioral intervention practices like exposure therapy for children with these kinds of experiences.

These findings should be interpreted in light of a complex background literature. Historically, many social skills training interventions failed to facilitate a measurable treatment effect for autistic youth (cf. Ferraioli & Harris, 2011). Yet, in recent years, numerous specific psychosocial treatments have exhibited positive, statistically significant effects on social responsiveness and peer engagement (e.g., DeRosier & Marcus, 2005; Frankel et al., 2010; Kasari et al., 2012; Laugeson et al., 2012; Storch et al., 2013; White et al., 2013; Wood et al., 2020, 2022), all of which utilize behavioral intervention components (e.g., modeling), and some of which utilize CBT, in several cases focusing specifically on autistic youth with maladaptive and interfering anxiety (e.g., Storch et al., 2013; White et al., 2013) as in the present study. The CBT interventions included in this set of studies explicitly focused on social skills, with overlap with some of the behavioral friendship training methods first introduced by Frankel et al. (2010), as well as components of CBT to support motivation, perspective taking, and skill acquisition. While it is sensible to be cautious in interpreting the findings from any given study, the aggregation of clinical trials with overlapping intervention features exhibiting signs of treatment response are an encouraging sign. These interrelated interventions may have a measurable effect on a meaningful target variable, social responsiveness.

Despite its positive track record in several formats, adapted CBT does not work for all autistic individuals. Rates of positive treatment response have varied substantially across studies (e.g., Storch et al., 2013; White et al., 2013; Wood et al., 2020), and there are likely differing trajectories of clinical benefit, even among participants who meet the same eligibility criteria. Similarly, a recent study employing latent profile analysis found that only a minority of participants experienced substantial clinical benefit (more than 50% symptom remission) in a large, national study of CBT treatment response among autistic adults presenting with impairing anxiety or depression in the United Kingdom (Pender et al., 2025). This analytic approach could be used beneficially to empirically identify latent classes of youth treatment response to adapted CBT, which could, in turn, facilitate research on factors predicting treatment response in this population. Ultimately, such research might be used to inform future improvements in interventions of this sort to better serve non-responders. For example, perhaps youth who are motivated to make closer social connections with others might benefit more from adapted CBT programs that emphasize social skill supports.

Some autistic stakeholders have persuasively noted that an alternative (and appealing) remedy for peer rejection is for others to adopt a more inclusive and affirming stance towards all peers (cf. Dwyer et al., 2024). For autistic youth who struggle with social responsiveness, unconditional social acceptance would likely address the hypothetical scenario described above in which peer rejection triggered or exacerbated mental health challenges. Structuring peer social contexts to foster greater inclusiveness (e.g., ensuring that recess group activities are varied and can be played by all students) is a pragmatic implementation of this concept that has had some success (e.g., Shih et al., 2019). Developing social skills that facilitate positive reception from peers is not incompatible with efforts to promote inclusiveness. Groups of all kinds have micro-cultures that are characterized in part by social engagement practices (cf. Corsaro & Eder, 1990; Fine, 1979), such as expectations about personal hygiene, assertiveness, aggression, and other characteristics that tend to impact others in the group. These practices help define how to ‘get along’ in a particular social group (e.g., McAdams, 2015) and may be seen as locally relevant social skills with likely implications for acceptance, friendship, and well-being. Social skill supports that appeal to intrinsic motivation (i.e., framing the skills as a means to achieve the person’s goals for friendship, feelings of belongingness, and fun) contrast favorably with supports framed within a deficit-oriented or conformity-focused rationale (e.g., these skills will help you “blend in” or “be like everyone else”). Given the range of desire for friendship and social participation among autistic individuals (Bennett et al., 2018, Chapter 9, pp. 173–189), many autistic individuals may view the effort to learn and practice social skills as worthwhile if they are offered in a neuroaffirming manner. In short, efforts to increase inclusion can be compatible with developmentally appropriate social skills interventions that build from intrinsic motivation.

Limitations of this study include the use of a mediator measured at post-treatment instead of during treatment. Although it is common in children’s mental health research for tests of potential treatment mechanisms to examine a target assessed at post-treatment (cf. McCart & Sheidow, 2016), a measurement timepoint that met the criteria for temporal precedence in the analysis of treatment mechanisms would have been ideal. Conclusions drawn from this study are also limited by the nature of the sample, which was less ethnically and racially diverse than the population from which it was drawn. Lastly, the sample only included children with at least moderate receptive and expressive verbal language skills, while children and youth on the autism spectrum have widely varying language skill profiles.

In further research, a study design permitting the analysis of temporal precedence (e.g., social responsiveness assessed repeatedly throughout acute treatment) could provide a more stringent and dynamic depiction of the hypothesized processes. A larger sample with greater diversity in terms of race, ethnicity, gender, and socioeconomic status would also be desirable to facilitate assessing for whom these mediation processes are applicable. Lastly, it would be important to include a more representative group of autistic youth in future studies of similar interventions to clarify whether social responsiveness mediates positive mental health and anxiety reduction among autistic youth with diverse language skills.

Strengths of this study include its relatively large sample size (for an RCT) and its use of measures with strong psychometric properties (as outcome measures) for autistic children and youth. Findings supported the hypothesis that social responsiveness may serve as a mediator of both anxiety outcomes and broader mental health treatment outcomes for an adapted CBT intervention that addresses social skills. Building relevant social skills as well as habituating to anxiety-provoking situations are both likely useful treatment components in CBT for autistic children and youth.
